# Is Radiotherapy a Risk Factor for Melanoma?

**DOI:** 10.3390/dermatopathology12040043

**Published:** 2025-11-17

**Authors:** Sumeyye Ozer, Priya Agarwal, Noah Musolff, Brendan Plann-Curley, Gizem Cosgun, Helen Yanyu Sun, Babar Rao

**Affiliations:** 1 Staten Island University Hospital at Northwell, Staten Island, NY 10305, USA; 2 Rutgers Robert Wood Johnson Medical School, New Brunswick, NJ 08901, USA; 3 Rao Dermatology, New York, NY 10007, USA; 4 Northwell, New Hyde Park, Long Island, NY 11040, USA; 5 Mehmet Akif Inan Hospital, Sanli Urfa 63300, Turkey; 6 Weill Cornell Medicine, New York, NY 10065, USA

**Keywords:** radiotherapy, melanoma, melanoma risk factors, melanoma secondary to radiotherapy

## Abstract

Melanoma is a highly aggressive skin cancer primarily linked to ultraviolet (UV) radiation. However, the potential role of ionizing radiation from radiotherapy in melanoma development remains unclear. This review synthesizes data from epidemiologic studies and case reports on melanoma after radiation exposure. Evidence indicates that childhood radiotherapy, even at low doses, is associated with an increased melanoma risk, plausibly reflecting the heightened radiosensitivity of developing melanocytes. Occupational radiation exposure, particularly in earlier eras with insufficient shielding, also appears to elevate risk. In patients exposed to radiation in adulthood, findings are mixed: large population datasets suggest a modest increase in melanoma following therapeutic radiation, whereas some case–control analyses do not demonstrate a clear dose–response relationship. UV radiation promotes melanomagenesis through direct DNA photoproducts driving characteristic C>T transitions at dipyrimidine sites, alongside oxidative stress and local immune modulation that facilitate malignant transformation. Collectively, individuals with prior radiotherapy, especially those irradiated in childhood, should be considered at increased melanoma risk and may benefit from long-term, targeted surveillance of irradiated fields. Awareness of this association between radiation exposure and melanoma may also support clinicopathologic correlation during the diagnostic evaluation of melanocytic lesions. Future work should define dose–response relationships in contemporary radiotherapy methods, characterize molecular signatures of ionizing radiation-associated melanomas, and establish evidence-based surveillance strategies for high-risk cohorts.

## 1. Introduction

Melanoma is a highly aggressive form of skin cancer originating from melanocytes, the pigment-producing cells of the skin [1]. Although melanoma represents only a small fraction of all skin cancer diagnoses, it is responsible for the majority of skin cancer-related deaths worldwide [2]. This disproportionate mortality is primarily due to melanoma’s high metastatic potential, enabling early dissemination to distant organs such as the lungs, liver, brain, and bones. When detected at an advanced stage, melanoma often shows a poor prognosis, with limited treatment options and lower survival rates compared to localized disease [2].

The primary and most widely studied environmental risk factor for cutaneous melanoma is exposure to ultraviolet (UV) radiation, either through cumulative low-dose exposure due to chronic sun exposure or intense, intermittent high-dose exposure such as severe, blistering sunburns. UV radiation induces DNA damage in melanocytes, leading to mutations in oncogenes and tumor suppressor genes that can initiate malignant transformation [3].

The process of UV-driven skin cancer formation is multifactorial. One pathway is direct mutagenesis, where UV light creates DNA lesions (cyclobutane pyrimidine dimers and 6–4 photoproducts) that lead to characteristic C>T transitions, known as the “UV signature.” Additionally, UV exposure generates reactive oxygen species, which cause indirect damage to the cell through oxidative DNA damage, lipid peroxidation, and protein modification [4]. This process induces local immunosuppression by depleting antigen-presenting cells and skewing cytokine profiles. It also disrupts critical melanocyte–keratinocyte signaling (e.g., the α-MSH–MC1R axis), which in turn promotes clonal expansion within a pro-inflammatory microenvironment. Together, these effects increase the probability of oncogenic hits in melanocytes and facilitate their progression to melanoma [4,5].

Electromagnetic radiation encompasses a broad spectrum of energy emitted from various sources, categorized into ionizing (e.g., X-rays and gamma rays) and non-ionizing radiation (e.g., radio waves, visible light, and UV radiation) based on its ability to remove electrons from atoms [6]. UV radiation, with wavelengths ranging from approximately 100 to 400 nm, is classified as non-ionizing radiation. In contrast, gamma radiation, commonly used in radiotherapy, has a much shorter wavelength and higher frequency, allowing it to penetrate deeper into tissues and exert ionizing effects. Both ionizing and non-ionizing radiation have the potential to cause mutagenic changes in cell structures, including proteins and DNA [6].

One of the significant long-term adverse effects associated with the administration of radiation therapy is the development of secondary malignancies, also referred to as radiation-induced cancers. These malignancies may arise within the high-dose region of the primary treatment field or its immediate periphery. Anatomical sites located at considerable distances from the irradiated area, although they receive minimal, low doses of scattered or stray radiation, can still contribute to carcinogenic risk over time. The occurrence of such secondary cancers underscores the importance of long-term surveillance in cancer survivors and highlights the need for continued efforts to optimize radiation delivery techniques to minimize exposure to healthy tissues [7].

Given the established role of non-ionizing UV radiation in melanoma pathogenesis, it is important to consider whether ionizing radiation, such as that administered during radiotherapy, might also contribute to melanoma development.

If a link between ionizing radiation exposure and melanoma exists, it would have significant clinical implications. Patients with a history of radiotherapy for other malignancies might benefit from enhanced dermatologic surveillance, particularly in areas of the body previously exposed to radiation. A greater understanding of the relationship between ionizing radiation exposure and melanoma risk could therefore guide further research in dermatopathologic diagnostic processes, research, as well as patient skin cancer screening guidelines.

## 2. Materials and Methods

To explore radiotherapy-induced melanoma, a comprehensive literature search was conducted in OVID Medline, Embase, and Web of Science. In addition to relevant MeSH and Embase terms, the search strategy included the following keywords: Melanoma* AND “following radiation” OR “following radiotherapy” OR “after radiation” OR “after radiotherapy” OR “prior* irradiat*” OR “previous* irradiat*” OR “radiation induced” OR “radiotherapy-induced” OR “radiation-associated” OR “radiotherapy-associated”.

Studies related to UV radiation-induced melanomas were filtered out of the databases using the Boolean NOT operator and the following terms:

“UV radiation” OR “UV Light” OR suntan* OR sunbath* OR sunscreen* OR ultraviolet OR “ultraviolet”.

During the selection process, studies focusing on UV radiation-induced melanomas were excluded. The review specifically targeted melanomas arising after ionizing radiation exposure located at the irradiated skin area. Additionally, recurrent melanomas arising after radiation treatment of primary melanomas were excluded.

## 3. Results

### 3.1. Radiation as a Risk Factor of Melanoma

#### 3.1.1. Radiation as a Risk Factor for Secondary Malignant Neoplasms

Ionizing radiation can cause cancer by damaging DNA, leading to breaks in one or both strands. Double-strand breaks are particularly dangerous, as they can cause mutations that drive cells to become cancerous. Single-strand breaks can also become double-strand breaks during cell division. Defects in DNA repair proteins, which normally detect and repair DNA damage, can heighten sensitivity to radiation and increase cancer risk [8].

The most extensive data for understanding radiation-induced cancer risk comes from long-term studies of atomic bomb survivors. These studies confirm that while second cancers after radiation therapy most frequently develop in tissues receiving high doses (over 2.5 Gy), a significant risk also exists in tissues exposed to much lower levels of radiation. Cancer risk is determined by several key factors, including the type of tissue exposed, the total and fractional radiation dose, the dose rate, and the time elapsed since exposure. Patients treated with fractionated RT, where radiation is given in multiple small doses totaling 15–50+ Gy, generally face lower cancer risks per unit dose than atomic bomb survivors who experienced a single large exposure. Most cancers caused by radiation emerge only after a long delay, often years or decades, and the associated cancer risk continues to increase as more time passes since exposure [8].

In a study comparing groups of patients treated with radiotherapy or not for breast cancer, elevated relative risks were observed for certain malignancies such as sarcoma, leukemia, gastric cancer, pancreatic cancer, bladder cancer, non-melanoma skin cancer, and kidney cancer [9]. Approximately two-thirds of secondary malignant neoplasms occur in the radiotherapy field [10]. The distinction between radiation-induced malignancy and a second primary neoplasm is important. To classify a malignancy as radiation-induced, several criteria must be satisfied: it must occur within the irradiated field, have a latent period preferably longer than four years, and have histology distinct from the primary neoplasm and compatible with a secondary tumor [10].

#### 3.1.2. Radiation Effects on Skin

Despite the rapid development and clinical adoption of advanced radiotherapy technologies aimed at improving precision, normal tissues are still inevitably exposed to radiation. One of the most common complications arising from this exposure is radiation-induced skin injury. The primary causes of radiation-induced skin injury include nuclear radiation accidents, occupational exposure, and most notably, tumor radiotherapy. The incidence of radiation-induced skin injury is steadily increasing, with studies indicating this common side effect affects approximately 85% to 95% of all patients undergoing radiotherapy [11].

Radiation-induced skin injury is generally categorized into two types: acute and chronic. Acute injury includes symptoms such as dry and wet desquamation, skin necrosis, ulcers, and bleeding. Chronic injury, on the other hand, encompasses conditions like chronic ulcers, radiation-induced keratosis, telangiectasia, fibrosis, and even skin cancer. Unlike skin injuries caused by other factors, radiation-induced skin injury is marked by a delayed onset, prolonged progression, and persistent symptoms.

Radiation causes direct and deep damage to the skin and underlying tissues, leading to dryness, loss of elasticity, pigmentation changes, soft tissue fibrosis, and radiation dermatitis. This process irreversibly harms the microvascular and endothelial cells, resulting in poor wound healing and increased susceptibility to infections [11]. Over time, this chronic tissue injury can progress to fibrosis via pathways like the TGF-beta 1 signaling cascade [12].

This carcinogenic environment is known to promote basal cell carcinoma (BCC), which arises from epidermal keratinocytes following oncogenic events in genes like PTCH1 or TP53 [13]. While the development of BCC is well-explained, the specific genetic pathways linking ionizing radiation to melanoma are less clear. However, emerging evidence from non-melanoma skin cancers (NMSCs) offers valuable clues. NMSC development is driven by systemic factors and shared carcinogenic pathways, including chronic inflammation and immune dysregulation [14]. A plausible mechanism emerges when considering that the initial damage from ionizing radiation—persistent inflammation, fibrosis, and vascular injury—mirrors the exact conditions known to promote NMSC.

These convergent processes, where radiation-induced damage may amplify inflammatory and immune circuits also involved in UV-driven carcinogenesis, support the need for vigilant surveillance of irradiated fields for both NMSC and melanoma.

#### 3.1.3. Melanoma Risk Factors

In the United States, melanoma is the fifth most common cancer in men and the sixth in women, with its incidence continuing to increase both nationally and globally [15]. Despite advancements in early detection and treatment, this upward trend underscores the importance of identifying and mitigating risk factors associated with melanoma development.

Notably, an estimated 90% of cutaneous melanomas can be attributed to the most significant modifiable risk factor: UV radiation exposure, including sunlight and artificial sources such as tanning beds [16,17]. Risk is increased by intense intermittent sun exposure, often resulting in sunburn, particularly in childhood or adolescence, as well as cumulative exposure over a lifetime [17].

Non-modifiable risk factors also play a role in the development of melanoma, such as for patients with fair skin tone, family history of melanoma, or genetic predisposition. Genetic predisposition is significant as 5–12% of melanoma cases are familial and often linked to high-penetrance mutations in genes such as CDKN2A, BAP1, and MC1R involved in DNA repair, immune function, cell adhesion, transcription, and melanin production. These variants can significantly increase melanoma risk, especially when combined with environmental UV exposures [16].

From an etiopathogenetic perspective, melanoma can be broadly divided based on its relationship to UV radiation. Melanomas arising on sun-exposed skin are associated with cumulative sun damage (CSD), which can range from low to high. Low-CSD melanomas, such as superficial spreading melanoma and nodular melanoma on intermittently sun-exposed areas like the trunk and extremities, typically occur in younger individuals. In contrast, high-CSD melanomas, including desmoplastic melanoma, lentigo maligna melanoma, and nodular melanoma on chronically sun-exposed areas such as the head, neck, and dorsal forearms, tend to develop in older adults [18].

On the other hand, certain melanomas show no apparent link to sun exposure, as they arise on sun-protected skin or sites not usually exposed to UV radiation. These include Spitz melanoma, acral melanoma (on palms, soles, and nail beds), melanomas arising from congenital or blue nevi, mucosal melanoma, and uveal melanoma [18].

#### 3.1.4. Evidence from Studies for Radiation-Associated Melanoma

Numerous studies have suggested a link between radiotherapy, whether therapeutic or occupational, and an increased risk of melanoma (Table 1). Notably, in a study evaluating radiologic technologists in the United States, melanoma risk was higher among technologists who began work before 1950 (RR = 1.8; 95% CI: 0.6–5.5), especially those with ≥5 years of work prior to 1950 (RR = 2.4; 95% CI: 0.7–8.7; *p* = 0.03), a period when occupational radiation exposure was likely greatest [19]. Additionally, those who did not regularly use lead shielding when they began working had a modestly increased risk (RR = 1.4; 95% CI: 0.8–2.5) [19].

In patients who received radiotherapy for cancer treatment, increased risk of melanoma was also observed. Among patients who received radioactive iodine for thyroid cancer, significantly elevated risks of melanoma of the head and neck were noted (SIR = 1.56; 95% CI: 1.22–1.97), particularly when the primary cancer was papillary thyroid (SIR = 1.69; 95% CI: 1.35–2.09) [20]. No increased risk was observed in patients who did not receive radioactive iodine [20]. Among childhood cancer patients, high-dose radiotherapy (>15 Gy) was associated with an increased risk of developing melanoma (OR = 13; 95% CI: 0.94–174) [21]. Notably, children treated for a gonadal tumor as a first malignant neoplasm were found to be at a higher risk of melanoma (OR = 8.7 (95% CI: 0.9–86)) [21]. Similarly, among children treated with radiation for skin hemangiomas, risk of melanoma was increased by about 3-fold (SIR = 3.0; 95% CI: 1.6–5.1), versus no increase in those not irradiated (SIR = 0.8) [22]. The risk was particularly elevated in patients treated with yttrium-90 (adjusted OR = 11.9; 95% CI: 1.4–123). Increased melanoma risk was observed even at low doses (<0.001 Gy) [22].

On a larger scale, an analysis of 520,977 breast cancer patients from the Surveillance, Epidemiology, and End Results (SEER) database (1973–2018) examined melanoma risk following radiation therapy [23]. The authors found that breast cancer patients who received radiation therapy demonstrated a higher risk of developing melanoma compared to those who did not (HR = 1.40; 95% CI: 1.30–1.51; *p* < 0.001). Melanoma incidence in these patients exceeded that of the general population in the United States (SIR = 1.12; 95% CI: 1.05–1.19; *p* < 0.05) [23].

Contrastingly, some studies refuted the link between radiation exposure and melanoma risk. A case–control study examining adulthood radiation exposure included 57 patients who developed melanoma after treatment for a first malignancy, matched with 171 controls [24]. Multivariate analysis found no significant association between radiotherapy and melanoma risk (OR = 1.01 per Gy; 95% CI: 0.96–1.07) [21]. The authors concluded that, unlike childhood radiation exposure, adult ionizing radiation exposure was not associated with increased melanoma risk [24].

In another study examining 98 patients who developed radiation-induced scalp malignancies following childhood irradiation for tinea capitis, the vast majority were diagnosed with basal cell carcinoma (n = 125) or squamous cell carcinoma (n = 16). Melanoma was identified in only one patient, who developed four lesions, highlighting its rarity and suggesting that, in contrast to other skin cancers, melanoma may be less frequently associated with ionizing radiation exposure to the scalp during childhood [13].

#### 3.1.5. Case Reports of Radiation-Associated Melanoma in the Literature

Between 1997 and 2017, numerous case reports documented malignant melanomas arising in diverse sites previously exposed to radiation. These locations included mucosal regions (such as the vagina, cervix, and esophagus), ocular areas (the conjunctiva, orbit, and eyelid), and various skin sites, including the scalp, irradiated burn scars, and breast skin post-radiotherapy (Table 2).

Affected patients included both males and females, ranging from childhood exposures to adult-onset treatments in their 40s through 60s. For instance, childhood scalp irradiation led to melanoma decades later, while middle-aged adults developed mucosal or cutaneous melanomas years after treatment (Table 2).

The latency period between radiation exposure and melanoma diagnosis was highly variable, ranging from 6 to over 40 years (Table 2). This wide range is consistent with the established latency for other radiation-induced solid tumors, which typically emerge from 20–40 years of post–exposure but can also manifest both earlier and later. In the specific case of neovaginal melanoma, it was diagnosed 16 years after radiotherapy and reconstruction [27]. Breast skin studies described synchronous angiosarcoma, melanoma, and morphea emerging 14 years post-treatment [36].

The diversity of radiotherapy regimens, from brachytherapy to external beam therapy, spanning doses from 15 Gy to 50 Gy, demonstrates that there is no clear threshold below which the melanoma risk is eliminated. Any ionizing radiation exposure to melanocytes appears to carry some carcinogenic potential. Notably, even low-dose radiotherapy, particularly when administered during childhood, is associated with a substantial long-term risk of melanoma development. Moderate-to-high therapeutic doses (40–50 Gy) used to treat malignancies consistently fall within the range linked to secondary melanomas. Moreover, fractionation schedules delivering larger doses per fraction may further increase carcinogenic potential compared to conventional fractionation of approximately 2 Gy per fraction. Collectively, these findings underscore the importance of lifelong dermatologic surveillance of previously irradiated fields, especially when cumulative doses exceed 15 Gy or when exposure occurred during childhood.

Causality was determined using three key criteria: location within the irradiated field, a latency exceeding four years, and histological confirmation of a new primary malignancy. Most reported melanomas met these standards, and this conclusion is reinforced by cases arising in sun-protected areas, which helps rule out UV exposure. Nevertheless, these case reports are prone to reporting bias and cannot be used to calculate incidence or define a dose–response relationship [8].

#### 3.1.6. Histologic Subtypes in Radiation-Associated Melanomas

Melanomas arising in irradiated areas show a wide variety of morphologic appearances and subtypes. The most reported subtype associated with radiotherapy is nodular melanoma, comprising 6 of 15 reported cases [25,26,27,30,31,32]. The second most common subtype is superficial spreading melanoma [29,34,38,39], followed by three mucosal/non-cutaneous melanomas and two desmoplastic melanomas [26,28,35,36,37].

More aggressive forms—such as nodular, desmoplastic, and mucosal melanomas—are often diagnosed at advanced stages in irradiated areas, with greater Breslow thickness and higher Clark levels [25,32,33]. In contrast, lentigo maligna melanoma, which is a less aggressive subtype typically associated with cumulative sun damage, has not been reported in irradiated regions (Table 1).

Atypical immunoprofile is also reported. For example, Rodriguez et al. reported an S100-negative mucosal melanoma arising in a prior radiation field. The diagnosis was supported by malignant epithelioid/spindle-cell morphology with pleomorphism and brisk mitoses; a corroborative melanocytic immunoprofile (SOX10, Melan-A/MART-1, and HMB-45 positivity); and exclusion of mimics via negative broad-spectrum cytokeratin (AE1/AE3), p63/EMA, and hematolymphoid markers. This spectrum, from typical markers to rare lineage-marker loss, should be considered when evaluating melanocytic tumors in irradiated tissues [28].

Rare primary mucosal melanomas, such as primary malignant melanoma of the esophagus composed of spindle-shaped and polygonal cells, underscore that radiotherapy-associated melanomas are not limited to cutaneous sites. Although mucosal melanocytes are rare, they can undergo malignant transformation following radiation, independent of ultraviolet exposure [26,28,37].

No molecular studies have been published specifically on radiation-associated melanomas. However, these melanomas predominantly present as nodular and superficial spreading types, which share similar mutational profiles.

### 3.2. Modern Radiotherapy Implications and Melanoma Risk

Contemporary RT techniques have transformed normal tissue sparing as compared to historical practices. Modern methods like image-guided IMRT/VMAT, stereotactic radiosurgery (SRS), stereotactic body radiotherapy (SBRT), and deep-inspiration breath-hold for thoracic/abdominal sites enable tighter margins, sharper dose fall-off, and improved conformality, thereby reducing high-dose exposure to skin and adjacent tissues. Dosimetric and epidemiologic studies in the modern era confirm lower out-of-field doses and a general decline in second solid cancer risks compared with earlier decades that employed larger fields and orthovoltage beams [7,40]. Proton therapy further reduces skin dose via the Bragg peak, though potential contributions from secondary neutrons require consideration and appear small with current delivery techniques [41].

Notwithstanding these advances, several uncertainties remain relevant to melanoma specifically. First, hypofractionated schedules and stereotactic techniques deliver a higher dose per fraction; while the integral dose to skin is typically reduced, the radiobiologic impact of these large fractions on melanocytes has not been fully delineated. Second, highly modulated photon techniques (e.g., VMAT/IMRT) can increase the volume of tissue receiving very low doses (the ‘low-dose bath’), with unclear implications for melanomagenesis [42,43]. Third, most contemporary cohorts lack the decades-long follow-up needed to detect radiation-associated melanomas, which often manifest after a long latency [43]. Therefore, while historical studies may overestimate current risk, they remain informative until robust, melanoma-specific, long-term data from modern RT cohorts become available.

Going forward, prospective registries should incorporate detailed skin dosimetry (including maximum and mean skin dose and low-dose volume metrics), treatment modality (IMRT/VMAT vs. 3D-CRT vs. protons), fractionation schema, and standardized long-term dermatologic surveillance. Such data will clarify whether technological advances have materially reduced radiation-associated melanoma risk and help refine surveillance recommendations for contemporary patients.

## 4. Discussion

This review highlights the complex relationship between ionizing radiation exposure and melanoma risk, synthesizing evidence from biological mechanisms, epidemiological studies, and case reports. While UV radiation, classified as non-ionizing electromagnetic radiation, remains the primary and most well-established environmental risk factor for cutaneous melanoma. This review focused on the potential role of ionizing radiation, particularly from therapeutic and occupational exposures, in melanoma development.

Mechanistically, ionizing radiation can directly damage DNA, causing both single-strand and double-strand breaks, the latter of which can result in mutations if not properly repaired. Deficiencies in DNA repair proteins exacerbate susceptibility to radiation-induced carcinogenesis [6]. The potential for development of secondary malignancies is a well-recognized long-term risk of radiotherapy. These cancers may develop within the high-dose radiation field but can also arise in distant tissues exposed to low doses through scattered radiation [7,8].

A growing body of evidence indicates a link between radiation exposure, whether therapeutic or occupational, and an increased risk of melanoma. Multiple studies show that childhood and early occupational exposure to ionizing radiation appear as significant risk factors [21,22]. Occupational exposure studies, such as the cohort of U.S. radiologic technologists, suggest elevated melanoma risk among those who began work before 1950, when occupational radiation doses were highest (relative risk up to 2.4), and among those who did not routinely use protective lead shielding [19]. These findings point to the potential hazard of chronic, unshielded exposure to low-dose ionizing radiation in the workplace, especially in earlier decades when safety standards were lacking.

Therapeutic radiation during childhood has been linked to an increased melanoma risk, particularly at higher doses, with some evidence of elevated risk even at lower exposures [21,22]. The elevated risk across a broad range of doses, including less than 0.001 Gy, suggests that developing melanocytes in children may be especially sensitive to radiation-induced oncogenic changes. However, confidence intervals in many of these studies are wide, and findings should be interpreted cautiously. Similar findings had been observed in secondary malignancies following radiotherapy for tumors other than melanoma. Additionally, cases of melanoma arising specifically within previously irradiated fields, with latency periods spanning decades, reinforce a localized, direct carcinogenic effect of radiation.

Conversely, adult therapeutic radiation presents more nuanced findings. In a case–control study of adults who developed melanoma after radiation for a first malignancy, no significant dose–response relationship was observed, suggesting that adult melanocytes may be less susceptible to radiation-induced transformation. Furthermore, a case–control study reported no increase in melanoma incidence among adults treated with radiotherapy, suggesting that melanoma risk may not increase substantially after lower-dose or adult-onset exposures [24]. Similarly, the cohort of patients irradiated for tinea capitis showed only a single case of melanoma among numerous basal and squamous cell carcinomas, underscoring the rarity of melanoma in this context [13]. These observations may reflect differences in tissue radiosensitivity between children and adults, variation in cumulative radiation dose, and limited statistical power due to the rarity of radiation-associated melanoma. Nonetheless, large-scale registry data from breast cancer survivors in the SEER database indicate a modest but significant increase in melanoma risk following radiotherapy, with risk exceeding the general population’s baseline [23]. This suggests that, at least in some adult populations, therapeutic radiation can contribute to melanoma risk, though the effect of size is smaller than observed with childhood exposure.

Moreover, among thyroid cancer patients, those treated with radioactive iodine, particularly for papillary thyroid cancer, showed increased risks of melanoma of the head and neck, while patients not receiving radioactive iodine did not show elevated risk. This points to a role for systemic radiation, such as radioactive iodine, in increasing melanoma risk at sites beyond the direct radiation field [20].

Importantly, case reports suggest that ionizing radiation may contribute to the development of mucosal melanomas in sun-protected tissues, including the vulva, uterine cervix, neovagina, and esophagus [26,27,37]. These cases are particularly informative because they arise in anatomical sites naturally shielded from sunlight, effectively excluding UV radiation, which is a well-established melanoma risk factor and a confounder. Mucosal melanomas, diagnosed years to decades after radiotherapy, suggest a possible link between local radiation exposure and subsequent malignant transformation of melanocytes within mucosal epithelium.

In addition to mucosal sites, case reports document malignant melanomas developing at various radiation-exposed sites: ocular and periocular melanomas in the conjunctiva, orbit, and eyelid skin; cutaneous melanomas on the scalp, burn scars or keloids, and on breast skin following radiotherapy for breast cancer (Table 2). For ocular melanomas arising after radiation therapy for retinoblastoma, genetic predisposition, such as retinoblastoma gene-1 (RB) mutations, can confound risk estimation [30,31]. However, case reports of melanomas arising in irradiated fields for benign conditions (e.g., tinea capitis, keloids) in patients without known cancer syndromes strengthen the argument for a direct carcinogenic effect of radiation. Hereditary RB predisposes individuals to a variety of new cancers over time, with radiotherapy potentially further enhancing the risk of tumors arising in the radiation field.

Focusing on pathogenesis, the degree of UV exposure influences the genomic profile of melanomas. For instance, increased UV radiation leads to a higher burden of point mutations and characteristic DNA changes, such as C to T transitions at dipyrimidine sites and, in some cases, T to A transversions, features known collectively as the “UV signature.” [5,18]. In future studies, molecular profiles of radiation-induced melanomas or non-melanoma secondary malignancies may contribute to revealing specific ionizing radiation signatures at the molecular level.

Latency periods varied widely, from as little as six years to over forty years. Patients affected ranged from children exposed to radiation at young ages to adults treated in their 40s–60s. For example, individuals who received scalp irradiation in childhood sometimes developed melanoma decades later, while middle-aged adults developed mucosal or cutaneous melanomas years after radiation therapy.

An unresolved question is whether melanomas within irradiated fields develop de novo or arise from preexisting melanocytic nevi. Large epidemiologic studies do not stratify by origin, limiting causal inference. In the cases in the literature we reviewed (n = 15), only one report explicitly documented melanoma associated with a preexisting congenital nevus, while the remainder did not identify a precursor, suggesting that de novo presentations may predominate [29]. This inference is tentative, as precursor lesions can be obscured by radiation-induced fibrosis, vascular damage, or pigmentary alteration. Prospective documentation of nevi in irradiated fields and careful histopathologic evaluation for residual nevus components is needed.

A separate study of patients who underwent childhood scalp irradiation for tinea capitis found numerous scalp tumors, predominantly basal cell and squamous cell carcinomas, with melanomas occurring only rarely [13]. This underscores the exceptional rarity of melanomas in irradiated scalp tissue compared to non-melanoma skin cancers. In addition, multiple case reports of melanomas arising after radiotherapy for tinea capitis further support the conclusion that radiation therapy, even when administered for benign conditions, can act as a risk factor for melanoma development.

Studies investigating the association between radiation exposure and melanoma risk exhibit significant methodological variability. Many are limited by imprecise exposure measurement and reliance on retrospective medical records. Additionally, they often fail to account for important confounding factors, including genetic predisposition, patterns of sun exposure, and concurrent therapies. Variability in study populations, radiation sources, dosage levels, and follow-up durations further hinders meaningful comparisons across studies. These challenges highlight the need for well-designed prospective studies that incorporate precise dosimetry to account for radiation exposure and thorough evaluation of potential confounders.

In the modern era of advanced molecular research, the pathogenesis of melanoma is increasingly understood through detailed genetic and mutational profiling. A particularly intriguing area of study might involve melanomas arising on skin exposed to ionizing radiation. Investigating specific genetic alterations and mutational signatures in these cases may help clarify the potential causal relationship between ionizing radiation and melanoma development.

Future research should focus on clarifying dose–response relationships for adult exposures and determining whether specific radiation doses or modalities influence melanoma risk. Studies exploring the molecular mechanisms by which radiation induces melanoma could identify biomarkers for susceptibility and targets for prevention. Further investigation is also needed into the interaction between inherited cancer predisposition syndromes and radiation exposure in melanoma risk. Additionally, research on the melanoma risk associated with modern, more precise radiation techniques could determine whether contemporary technologies mitigate risk. Prospective studies assessing optimal surveillance strategies would help establish evidence-based guidelines for skin cancer monitoring in radiation-exposed patients.

## 5. Conclusions

The available evidence suggests an increased risk of melanoma following childhood radiotherapy, particularly after high-dose or targeted treatment, though studies are limited and wide confidence intervals present statistical uncertainty. There is also moderate evidence linking earlier occupational radiation exposure to a higher melanoma risk. While adult therapeutic radiation appears to confer a small increase in risk, this association has not been consistently statistically significant across all studies. Importantly, cases of melanomas developing in sun-protected mucosal and skin sites within irradiated areas, sometimes many years after exposure, support the idea that ionizing radiation itself is a direct cause of melanoma, independent of UV light.

These findings underscore the importance of lifelong skin monitoring for individuals, particularly those treated in childhood or exposed to substantial occupational radiation. Dermatologic follow-up should be part of survivorship care plans, with routine skin exams by dermatologists or trained providers, focusing on previously irradiated regions. Furthermore, for dermatopathologists, knowledge of a patient’s radiation history provides a crucial clue during the clinicopathologic correlation of melanocytic lesions. Patient education—informing patients about melanoma signs, encouraging regular self-exams, and promoting sun protection—may further lower the risk. Meanwhile, strict adherence to radiation-safety protocols, including proper shielding and accurate dosimetry, remains essential to reduce occupational exposure. For adult patients receiving radiotherapy, clinicians should be aware of the small but real melanoma risk, balancing vigilance with the need to provide necessary cancer treatment.

For the future, by integrating molecular data with clinical and histopathological findings, the mechanisms of radiation-induced melanomagenesis can be better delineated, potentially leading to improved diagnostic accuracy, risk stratification, and targeted therapeutic strategies for affected patients. In addition, prospective studies with precise radiation-dose assessment and adequate statistical power are needed to quantify melanoma risk more definitively and to inform evidence-based prevention and monitoring strategies. Increased awareness among both clinicians and patients will help ensure earlier detection and improved outcomes for those at elevated risk of radiation-associated melanoma.

## Figures and Tables

**Table 1:** Studies investigating melanoma diagnosis in patients with prior radiotherapy history.

Study	Enrollment	Type of Radiation	Melanoma Cases	Outcomes
Freedman et al. [19]	68,588	X-ray	207	Increased melanoma risk among X-ray technologists who began work before 1950 (RR = 1.8; 95% CI: 0.6–5.5) Increased melanoma risk among those who did not regularly use lead shielding (RR = 1.4; 95% CI: 0.8–2.5)
Rezaei et al. [20]	174,916	Various	790	Increased melanoma risk of the head and neck with radiotherapy for thyroid cancer (SIR = 1.56; 95% CI: 1.22–1.97)
Guerin et al. [21]	29,521	External beam	16	High-dose radiotherapy (>15 Gy) was associated with an increased risk of developing melanoma for childhood cancer patients (OR = 13; 95% CI: 0.94–174)
Haddy et al. [22]	4620	Radium applicators, pure β-emitters, and contact X-ray	13	Increased melanoma risk (SIR = 3.0; 95% CI: 1.6–5.1), particularly with yttrium-90 for childhood skin hemangiomas (adjusted OR = 11.9; 95% CI: 1.4–123)
Luo et al. [23]	520,977	Unspecified	1876	Increased melanoma risk among breast cancer patients treated with radiation therapy (HR = 1.40; 95% CI: 1.30–1.51; *p* < 0.001).
Dupuy et al. [24]	228 (including 171 matched controls)	External beam therapy	57	No significant association between radiotherapy and melanoma risk in adult cancer patients (OR = 1.01 per Gy; 95% CI: 0.96–1.07)
Maalej et al. [13]	98	External beam, brachytherapy, and/or combined treatment	4	Most cases were basal cell carcinoma (*n* = 125) or squamous cell carcinoma (*n* = 16) Melanoma was identified in only one patient, who developed four lesions

**Table 2:** Case reports of radiation-associated melanomas in the literature.

Age	Sex	Primary Tumor	Melanoma Site	Interval (Years)	Melanoma Subtype & Morphology	Type of Radiation	Reference
68	F	Cervical squamous cell cancer	Labium majus	4	Nodular melanoma Small cells, Clark IV	External beam radiotherapy and brachytherapy	[25]
73	F	Squamous cell carcinoma	Cervix and vaginal fornix	9	Nodular Melanoma	External beam RT 40 Gy in 12 fractions over 4 weeks	[26]
71	F	Squamous cell carcinoma	External Neovagina	16	Nodular Melanoma Epithelioid cells with vacuolated cytoplasm	4500 cGy in 25 fractions to the neovagina	[27]
41	F	Childhood rhabdomyosarcoma	Nasal mucosa	30	Melanoma in the orbit S-100 negative		[28]
17	M	Astrocytoma	Head	10	Superficial spreading melanoma Associated with congenital nevi		[29]
40	F	Retinoblastoma	Left eye	30	Melanoma in the orbit Heterogeneous mix of spindled to epithelioid cells	Enucleation and external beam radiation therapy	[30]
4	NA	Retinoblastoma	Eye	3	Nodular melanoma Amelanotic		[31]
64	M	Tinea capitis	Scalp	50	Nodular Melanoma Breslow thickness 9.5 mm, Clark V	X ray	[32]
75	M	Tinea capitis	Scalp	>30	Nodular Melanoma Breslow thickness 5.6, Clark IV	Radiotherapy	[33]
53	F	Ankylosing spondylitis	Spine skin	30	Superficial Spreading Melanoma in situ	Radiotherapy, a total of 1500 cGy (a standard dose)	[34]
61	F	Burn scar	Arm	>50	Desmoplastic Melanoma	Low-dose radiation therapy	[35]
57	M	Keloid	Chest	48	Desmoplastic melanoma	Low-dose external beam irradiation	[36]
74	M	Squamous cell carcinoma	Esophageal mucosa	6	Primary malignant melanoma of the esophagus Spindle-shaped and polygonal cells	Radiotherapy, with 50 Gy/25 Fr	[37]
68	F	Breast Cancer	Breast skin	14	Superficial spreading melanoma in situ	50 Gy, 36 fractions in 3 months Radiotherapy	[38]
78	F	Meningioma	Periocular skin	32, 22	Superficial spreading melanoma Breslow thickness, 0.6 mm 2 additional foci of melanoma in situ	Radiotherapy	[39]

## Data Availability

No new data were created or analyzed in this study.

## Abbreviations

The following abbreviations are used in this manuscript:

F	Female
M	Male
NA	Not Applicaple
UV	Ultraviolet
BCC	Basal cell carcinoma
CSD	Cumulative sun damage
SEER	Surveillance, Epidemiology and End Results
RB	Retinoblastoma
OR	Odds Ratio
RR	Relative Risk
SIR	Standardized incidence ratio
IMRT	Intensity-Modulated Radiation Therapy
VMAT	Volumetric Modulated Arc Therapy
SRS	stereotactic radiosurgery
SBRT	stereotactic body radiotherapy
